# Both inhibitory and activating KIRs recognize RIFINs: a dual-edged mechanism of NK cell control in malaria

**DOI:** 10.1038/s41392-025-02341-5

**Published:** 2025-08-13

**Authors:** Angelica Cuapio, Hans-Gustaf Ljunggren

**Affiliations:** Center for Infectious Medicine, Department of Medicine Huddinge, Karolinska Institutet, Stockholm, Sweden

**Keywords:** Infectious diseases, Innate immune cells

In a recent publication in *Nature*, Sakoguchi et al. reveal a long-sought missing link between pathogen-derived ligands and activating human natural killer (NK) cell receptors.^1^ The study identifies a clade of *Plasmodium falciparum* (*P. falciparum*) repetitive interspersed family (RIFIN) proteins that not only bind to the inhibitory KIR2DL1 receptor but, strikingly, also engage the activating KIR2DS1 receptor, thereby offering new insight into NK cell regulation in malaria and expanding our understanding of host–pathogen interaction in innate immune responses.

NK cells are key effectors of the innate immune response, equipped with a repertoire of inhibitory and activating receptors that finely balance immune activation. Among these are the killer cell immunoglobulin-like receptors (KIRs), which exist in paired forms: inhibitory KIRs (e.g., KIR2DL1) signal tolerance through immunoreceptor tyrosine-based inhibition motifs, whereas activating KIRs (e.g., KIR2DS1) signal activation via association with adapter proteins like DAP12. While inhibitory KIRs are well established as recognizing human leukocyte antigen (HLA) class I molecules and mediating NK cell tolerance to self, the biological significance of activating KIRs has remained comparably more elusive.^2,3^ The findings presented by Sakoguchi et al. offer the first experimental demonstration of a pathogen-derived ligand for an activating KIR that is not derived from or presented by major histocompatibility complex (MHC) class I molecules, providing a molecular rationale for their evolutionary retention.^1^

**Fig. 1:**
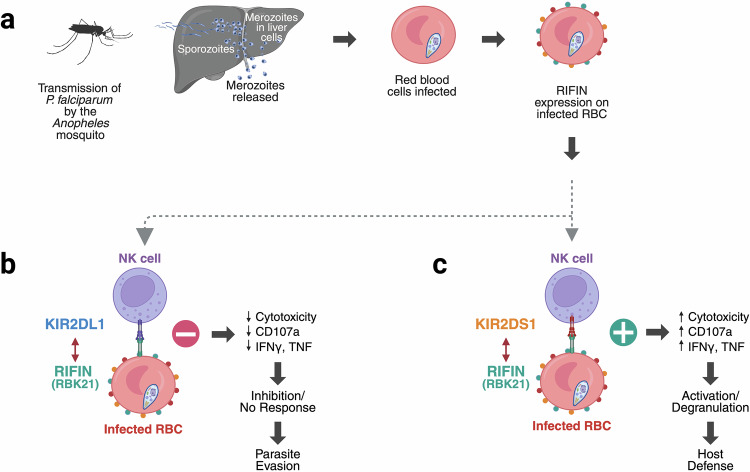
RIFINs engage both inhibitory and activating KIRs to modulate the NK cell response during P. falciparum infection. **a** Representative life cycle of the *P. falciparum*, from mosquito *Anopheles* transmission, followed by hepatic parasite replication, release of merozoites into blood circulation, erythrocytic infection, and surface expression of RIFIN molecules on infected RBCs. A conserved clade of KIR2DL1-binding RIFINs, named RBK21, is found in different field strains from both African and Asian regions. **b** Engagement of KIR2DL1 to RBK21 induces NK cell inhibition, reducing degranulation and cytokine production, consequently promoting immune evasion by the parasite. **c** RBK21 can also bind to the activating receptor KIR2DS1, which triggers NK cell activation, enhancing degranulation and cytokine production, thereby promoting host defense. These findings unveil an MHC class I-independent microorganism-derived ligand for an activating KIR indicative of a host–parasite co-evolution in shaping immune responses. Illustration generated with BioRender.com

RIFINs, encoded by a large multigene family in *P. falciparum*, are known virulence factors expressed on the surface of infected red blood cells (RBCs) (Fig. 1a). Prior studies have shown that some RIFINs bind inhibitory immune receptors such as LILRB1 and LAIR1, suppressing NK cell activation and favoring parasite survival.^4^ In their study, Sakoguchi and colleagues systematically screened RIFIN expression libraries from both laboratory-adapted and field-isolated strains of *P. falciparum* across Africa and Asia. They identify a distinct clade of RIFINs, representing about 10% of the parasite’s ~150-member RIFIN family, that exhibit high-affinity binding to the inhibitory receptor KIR2DL1.^1^

These KIR2DL1-binding RIFINs suppress NK cell activation in vitro, confirming their functional role in immune evasion (Fig. 1b). Structural studies of two representative RIFINs (RBK21 and KEN-01) in complex with KIR2DL1 reveal a conserved binding interface centered on a hydrophobic core stabilized by an intermolecular β-sheet, providing a mechanistic understanding of the high-affinity interaction. Importantly, this structural interface is largely preserved in KIR2DS1, the activating counterpart of KIR2DL1, prompting the authors to test whether these RIFINs could also engage KIR2DS1. Indeed, they demonstrate that RIFINs capable of binding KIR2DL1 can also trigger functional activation of KIR2DS1-expressing NK cells in a DAP12-dependent manner, resulting in degranulation (CD107a expression) and cytokine production (interferon-γ and tumor necrosis factor) (Fig. 1c). This evidence of NK cell activation offers a paradigm shift: the same parasite protein can serve both as an immunoevasive ligand and an activator of an immune response, depending on the receptor context.^1^

Since RBCs lack MHC class I expression, the involvement of both inhibitory and activating KIRs in recognizing *P. falciparum*-infected erythrocytes is particularly striking. Under normal circumstances, NK cell recognition of RBCs is limited and, when it occurs, it is thought to be primarily mediated through antibody-dependent cellular cytotoxicity, wherein NK cells are recruited via Fc receptor engagement following opsonization. The dual functionality of RIFINs therefore opens new perspectives on NK cell recognition of infected RBCs. While many viruses, including cytomegalovirus and human immunodeficiency virus, downregulate specific MHC class I molecules to evade T cell detection and exploit inhibitory KIRs to dampen NK cell responses towards MHC class I-expressing target cells, this study is the first to show a pathogen-evolving ligand that, independently of MHC class I, engages both inhibitory and activating KIRs. One plausible explanation is that *P. falciparum* leverages this unusual strategy of molecular mimicry to fine-tune the host response in a stage- and/or context-dependent manner.

The identification of KIR2DL1-binding RIFINs in both Southeast Asian and African field strains underscores the evolutionary conservation of this immune evasion strategy, suggesting selective pressure to preserve this mechanism across *P. falciparum* populations. From an evolutionary standpoint, the ability of a pathogen to exploit both sides of the KIR signaling axis reflects a sophisticated adaptation to human immunity.

Functionally, these findings also have implications for understanding inter-individual variability in malaria susceptibility. KIR genotypes vary significantly among human populations, and the presence or absence of certain KIR/HLA combinations has been associated with differential malaria outcomes.^5^ This study suggests that RIFIN expression and KIR repertoires have co-evolved to fine-tune the host–parasite interface. Populations with high frequencies of activating KIRs such as KIR2DS1 may exhibit enhanced NK cell activation and parasite clearance, while those dominated by inhibitory KIRs may be more susceptible to RIFIN-mediated immune suppression. The therapeutic implications are equally intriguing. Blocking RIFIN–KIR2DL1 interactions could release NK cell inhibition and restore anti-parasitic activity. Conversely, enhancing RIFIN–KIR2DS1 signaling might potentiate innate immune activation. These strategies could complement vaccine or antibody-based approaches that target other parasite antigens.

In conclusion, Sakoguchi et al. have uncovered a major new layer of immunological insight in the malaria–NK cell axis by identifying a conserved class of parasite proteins that function as ligands for both inhibitory and activating KIRs. This discovery not only resolves a key missing link in the biology of activating KIRs but also lays the groundwork for future studies exploring immune modulation and therapeutic intervention targeting NK cell responses in malaria and potentially other infectious diseases.
